# Predicting Response to Treatment and Survival in Advanced Ovarian Cancer Using Machine Learning and Radiomics: A Systematic Review

**DOI:** 10.3390/cancers17030336

**Published:** 2025-01-21

**Authors:** Sabrina Piedimonte, Mariam Mohamed, Gabriela Rosa, Brigit Gerstl, Danielle Vicus

**Affiliations:** 1 Division of Gynecologic Oncology, Hospital Maisonneuve Rosemont, University of Montreal, Montreal, QC H3T 1J4, Canada; 2 Faculty of Medicine, University of Montreal, Montreal, QC H3T 1J4, Canada; mariam.mohamed@umontreal.ca; 3 The Rosa Institute, Melbourne, ACT 2001, Australia; gabriela@naturalfertilitybreakthrough.com (G.R.); brigit@naturalfertilitybreakthrough.com (B.G.); 4 Division of Gynecologic Oncology, Sunnybrook Health Sciences Center, Toronto, ON M4N 0A4, Canada; danielle.vicus@sunnybrook.ca

**Keywords:** ovarian cancer, machine learning, radiomics, treatment prediction

## Abstract

**Background and Objective:** Machine learning and radiomics (ML/RM) are gaining interest in ovarian cancer (OC) but only a few studies have used these methods to predict treatment response. The objective of this study was to review the literature on the applications of ML/RM in OC assessments, specifically focusing on studies describing algorithms to predict treatment response and survival. **Methods:** This is a systematic review of the published literature from January 1985 to December 2023 on the use of ML/RM in OC An extensive search of electronic library databases was conducted. Two independent reviewers screened the articles initially by title then by full text. Quality was assessed using the MINORS criteria. *p*-values were generated using the Pearson’s Chi-squared (x^2^) test to compare the performances of ML/RM models with traditional statistics. **Results:** Of the 5576 screened articles, 225 studies were included. Between 2021 and 2023, 49 studies were published, highlighting the rapidly growing interest in ML/RM. Median-quality scores using the MINORS scale were similar between studies published between 1985–2021 and 2021–2023 (both 8). Neural Networks (22.6%) and LASSO (15.3%) were the most common ML/RM algorithms in OC. Among these studies, 13 focused specifically on prediction of treatment response using radiomics. A total of 5113 patients were analyzed. The most common algorithms were Random Forest (4/13) followed by Neural Networks (3/13) and Support Vectors (3/13). Radiomic analysis was used to predict response to neoadjuvant chemotherapy in seven studies, with a median AUC of 0.77 (range 0.72–0.93), while the median AUC was 0.82 (range 0.77–0.89) in the six studies assessing the prediction of optimal or complete cytoreduction. Median model accuracy reported in 7/13 studies was 73% (range 66–98%). Additionally, four studies investigated the use of ML/RM for survival prediction for OC. The XGBoost model had 80.9% accuracy in predicting 5-year survival compared to linear regression, which achieved 79% accuracy. The Random Forest model has 93.7% accuracy in predicting 12-month progression-free survival, compared to 82% for linear regression. **Conclusions:** In conclusion, we found that the use of ML/RM algorithms is becoming a more frequent method to predict responses to treatment of OC. These models should be validated in a prospective multicenter trial prior to integration into clinical use.

## Simple Summary

The study initially presents an overview of the literature on studies using machine learning in ovarian cancer. The most common algorithms are neural networks. Since this topic is exceedingly vast, we then focus on studies reporting the use of machine learning and radiomics to predict treatment response, surgical outcome, and survival. For the prediction of the response to neoadjuvant chemotherapy, most studies had a performance around 80%, which is higher than usual statistics; these studies extracted data from clinical parameters and radiology, and may help select patients for surgery. In patients undergoing surgery, the models described in the study had a performances also over 80%. Furthermore, the highest performing model was in a platinum-resistant setting with an AUC > 95%, which can be particularly clinically useful to select patients for novel targeted therapies such as *mirvetuxumab*. We also summarize clinical biomarkers that may be used to predict survival. Thus, the role of ML and radiomics in ovarian cancer is very promising to improve the prediction of treatment outcomes and to assist in decision making for optimal treatment modality to ultimately improve survival. Future prospective studies are needed to confirm these findings; however, our study presents the first overview of the available literature.

## 1. Introduction

Ovarian Cancer (OC) is the leading cause of death from gynecologic malignancy [1,2,3]. Improvements in survival in the recent decades have focused on maximal cytoreductive effort, intraperitoneal chemotherapy, Heated Intraperitoneal Chemotherapy (HIPEC), and, most recently, on the introduction of PAR inhibitor maintenance therapy [4,5]. An important challenge still remains in the selection of suitable candidates for neoadjuvant chemotherapy (NACT) versus primary cytoreductive surgery. This decision is crucial to offer maximal survival while minimizing surgical complications. Several models exist to predict the outcomes of cytoreduction, but these are limited by traditional statistics, data linearity, and only a finite number of variables [6,7,8,9,10]. Recently, objective tools have been devised to predict response to NACT, but harbour similar limitations to traditional statistics [11]. Thus, more accurate prediction strategies are needed to offer patient-centred, individualized treatment.

Machine learning (ML) has emerged as a better prediction tool in medicine than traditional statistics, as it can overcome the limitations of data linearity and identify patterns within data, allowing for more accurate assessments using algorithms known as classifiers [12]. ML models can handle multiple factors simultaneously, and thus can be particularly useful to identify biomarkers, observe the patterns within preoperative diagnostic imaging and clinical data used to predict responses to treatment and survival. ML is gaining popularity within other fields of medicine, such as in general surgery, but has yet to be used in gynecologic oncology, where the current need for improved precision and patient selection is pressing [13,14,15]. Furthermore, recognizing patterns within diagnostic images through the field of radiomics can lead to enhanced prediction capability, as it extracts a high volume of data from medical imaging and uses heterogenic features to provide an objective, improved prediction through mathematical analysis [16,17].

To date, only few studies have explored the use of ML and radiomics in advanced ovarian cancer. We sought to perform a systematic review on all machine learning algorithms used in ovarian cancer; this was previously published as an abstract [18]. We found that between 1985 and 2022, there were 85 articles published, and this increased threefold in the last decade. These studies focused mostly on clinical datasets (33%), imaging (30.7%), biomarkers (22%), and genomics (12.5%). The most commonly used algorithms were support vector machines (28%) and neural networks (25.3%). However, the data are very extensive when describing all aspects of patient care, and heterogeneity in data precludes the reporting of a meta-analysis. Thus, for clinical significance, the focus of this paper was specifically on the prediction of treatment outcomes and survival in advanced ovarian cancer.

The objective of this study was twofold: to present a broad overview on the applications of ML/RM in OC describing the most frequent algorithms used and summarizing the performances of the models. Secondarily, this analysis focuses specifically on studies that predict the response to treatment (surgery and chemotherapy) and that determine their impact on improving survival.

## 2. Materials and Methods

**Figure 1:**
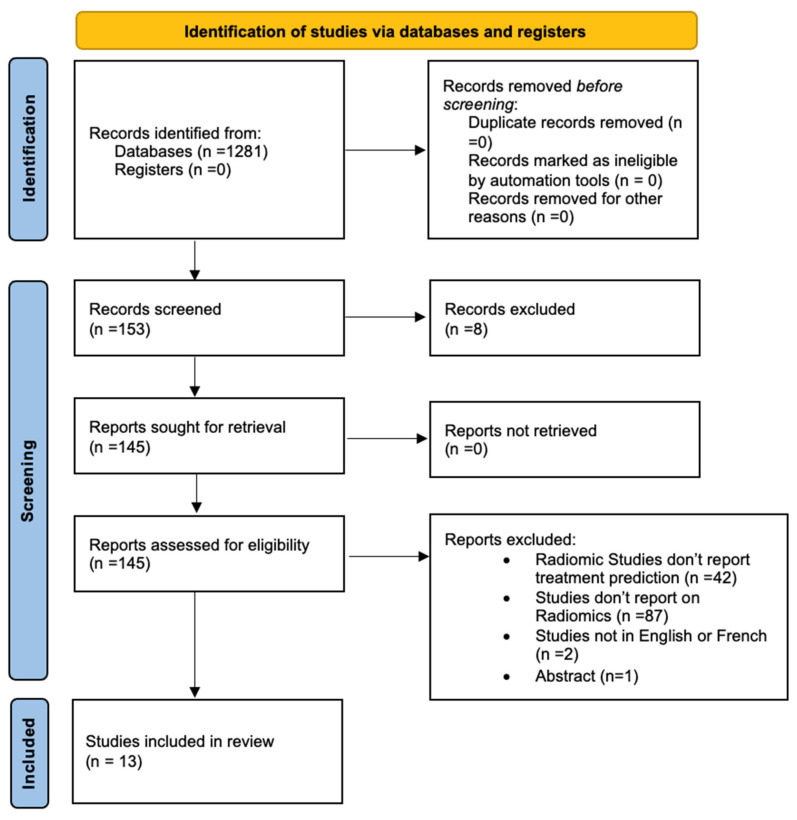
PRISMA flow diagram of the study selection process.

A systematic review was conducted following Preferred Reporting Items for Systematic Reviews and Meta-Analyses (PRISMA) guidelines [19] (Figure 1). The protocol was registered in PROSPERO (CRD42021269565).

### 2.1. Search Strategies and Screening Processes

An electronic database was performed in PubMed and Google Scholar in January 2024. The following medical search terms were used, as well as their combination: “Genital Neoplasms, Female” OR “Peritoneal Neoplasms” OR “Ovarian Neoplasms” OR “Gynecologic” OR “Ovarian” OR “Ovary” OR “Ovaries” OR “Peritoneal fallopian tube” OR “Ovarian cancer” OR “Ovarian neoplasm” OR “Ovarian malignant” or “Ovarian carcinoma” AND “Artificial Intelligence” OR “Machine Learning” OR “Deep Learning” OR “Unsupervised Machine Learning” OR “Neural Networks, Computer” OR “Artificial Intelligence” OR “AI” OR “unsupervised learning” OR “ensemble method*” OR “complex neural network*.mp.” Study selection was limited to articles in English and French. Duplicates were removed before title and abstract screening.

### 2.2. Eligibility Criteria

We have excluded articles that were conference abstracts, non-English or French studies, animal studies, and other non-relevant studies. Our inclusion criteria focused on full text articles published since March 1985 in English or French and articles that used ML models to predict OC outcomes, namely treatment, recurrence, and/or survival.

### 2.3. Study Selection, Data Extraction, and Statistical Analysis

Articles were initially screened based on title and abstract by one of the authors (M.M.). The final list of eligible articles was then reviewed and selected by two authors (M.M. and S.P.). Any discrepancies were resolved between reviewers through discussion until a consensus was reached. Data were then extracted and compiled in an Excel sheet for analysis. Based on agreement, we extracted data on study characteristics and features: author, title, type of publication, country of origin, year, the objectives of the study, study design, inclusion, and exclusion criteria; the type of treatment received as well as intervention and outcomes like type of ML applied; and accuracy, precision, sensitivity, specificity, diagnosis, gene identification, serum, and survival. The outcomes extracted from each study were accuracy, specificity, and sensitivity. These were reported based on the training, validation and tests sets. Means were reported if possible. Data were analyzed using a combination of Microsoft Excel and Google sheets. This paper focuses specifically on the prediction of treatment response, as the entire topic of ovarian cancer and ML would be too vast and require multiple publications.

### 2.4. Definitions of Machine Learning Models

Random Forest is an ensemble method that draws K bootstrap samples (B_1_–B_k_) with replacements and constructs K trees (T_1_–T_k_) from these [12]. The resulting model is the average estimate and has no need for smaller decision trees. A gradient-boosted tree is a tree-based method that builds on a series of smaller trees based on weighted majority; more accurate trees have more weight, and subsequent trees based are on predictions missed in previous trees [12]. The final model is a weighted sum of the individual trees.

Neural Networks are ML models similar to the neurons of a human brain. They consist of layers of nodes, or artificial neurons: an input layer, one or more hidden layers, and an output layer. Each node connects to others, has its own associated weight and threshold, and makes predictions or connections for a specified outcome [12]. This algorithm is commonly used to make predictions using images.

The Least Absolute Shrinkage and Selection Operator (LASSO) model is a regression analysis method that performs both variable selection and regularization in order to enhance prediction accuracy and interpretability [20]. A Support Vector Machine (SVM) is a supervised machine learning algorithm that classifies data by finding an optimal line or plane that maximizes the distance between each class in N dimensions, which can maximize the points between decision boundaries and is commonly used.

Area Under the Curve (AUC) is a commonly used measure of prediction. A number closer to 1 maximizes sensitivity and specificity, thus highlighting a better prediction performance as compared to those with lower values.

### 2.5. Method Quality Assessments

A methodological index for non-randomized studies (MINORS) was used to assess the risk of bias of the included studies [21].

## 3. Results

**Figure 2:**
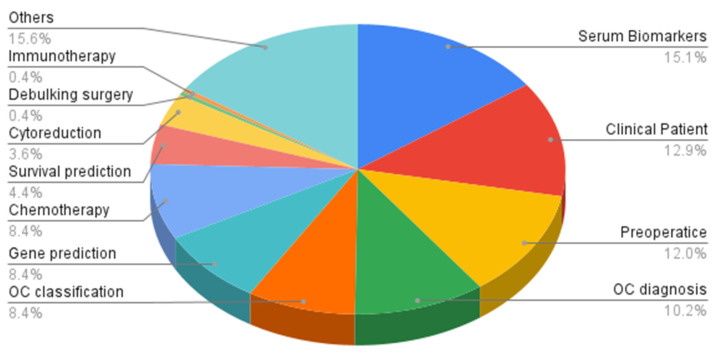
Common applications of machine learning in ovarian cancer among 224 studies.

**Figure 3:**
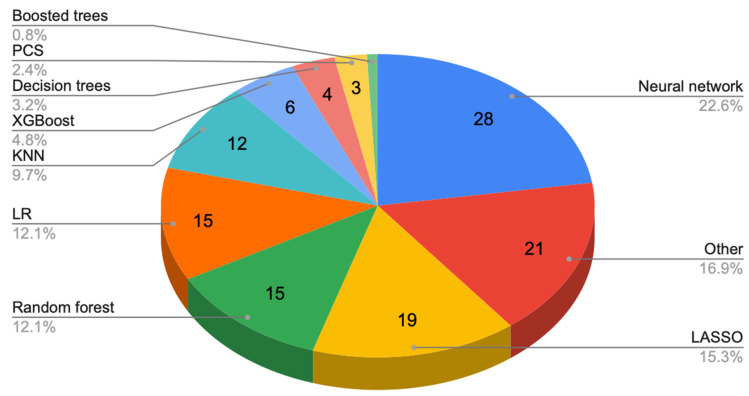
Pie chart of all ML techniques described in the included studies (*n* = 51) (others: UA&MLR, CART, Elastic Net, naïve Bayes (NB), multi-layer perceptron (MLP), MLDTA, gradient-boosting machines (GBMs), MAC-Net, ELM, LDA, ResNet, Cox regression, SGD, DSS, MVA, K-mean clustering, and SE-SPP-DenseNet).

Of the 5576 screened articles on machine learning in ovarian cancer, 225 studies were included. Between 2021 and 2023, 49 studies were published, highlighting the rapidly growing interest in ML/RM. There were 34 (15.1%) studies on serum biomarker development, 29 (12.9%) using clinical patient datasets, 27 (12%) on preoperative diagnosis, 23 (10.2%) on OC diagnosis, 19 (8.4%) on OC classification, 19 (8.4%) studies assessing gene prediction, 19 (8.4%) on chemotherapy response prediction, 10 (4.4%) on survival prediction, 9 (4%) on prediction of cytoreduction, 1 (0.4%) on immunotherapy response prediction, and 35 (14.7%) others (Figure 2). Median-quality scores using the MINORS scale were similar between studies published between 1985–2021 and 2021–2023 (MINORS Score 8). Neural Networks (22.6%) and LASSO (15.3%) were the most common ML/RM algorithms used in OC (Figure 3).

Among these studies, 13 focused specifically on prediction of treatment outcomes, cytoreduction, or survival (Table 1). A total of 5113 patients were included in these studies. The most common algorithms were Random Forest (4/13, 30.7%) followed by Neural Networks (3/13, 23.1%) and Support Vectors (3/13, 23.1%). Radiomic analysis was used to predict response to neoadjuvant chemotherapy in seven studies, with a pooled median AUC of 0.77 in the test set (range 0.72–0.93). The outcome of the prediction models was a complete response on imaging. In the six studies assessing the prediction of optimal or complete cytoreduction, the pooled median AUC was 0.82 in the test set (range 0.77–0.89). The median model accuracy reported in 7/13 studies was 73% (range 66–98%).

Additionally, four studies investigated the use of radiomics for survival prediction in OC. The model called *XGBoost*, which consisted of boosted trees, had 80.9% accuracy in predicting 5-year survival compared to linear regression, which achieved 79% accuracy. The Random Forest model had 93.7% accuracy in predicting 12-month progression-free survival, compared to 82% for linear regression.

## 4. Outcome Prediction

### 4.1. Response to Neoadjuvant Chemotherapy

Rundo et al. [22] assessed computed tomography (CT) radiomics to predict response to NACT using a pathologic chemotherapy response score (CRS) during surgery as an outcome. The CRS is a three-tiered pathological system related to responses to chemotherapy on surgical specimens of the omentum in interval cytoreductive surgery. Based on a sample of 109 HGSOC patients comparing CRSs on the omentum, pre-NACT, and post-NACT, the AUCs of the model were 0.68 and 0.87, respectively, for predicting complete responses to imaging. The addition of radiomics extracted from CT images improved the NPV significantly (*p* < 0.0001) and reduced PPV variability. The radiomics model, incorporating five frequently selected features, provided superior AUCs of 0.75 ± 0.04 in the discovery set and 0.68 ± 0.03 in the external test set, suggesting a more accurate predictive value of CT radiomics for complete responses to NACT, as evidenced by a pathologic CRS of 3.

Crispin-Ortuzar et al. [23] also developed an integrative radiogenomic framework to predict responses to NACT, incorporating data from clinical features, chemotherapy treatment details, CA-125 levels, circulating tumour DNA, and radiomics features extracted from pre- and post-NACT CT scans. The model accurately predicted radiologic tumour shrinkage (at least 30% based on RECIST criteria), *p* = 0.04. There were 124 patients used to created the model. The integrated radiomics model had greater prediction of treatment response with a Spearman’s correlation of 0.5, *p* = 0.02, compared to models that lacked the integration of radiomics. The model presented a superior AUC of 0.78 versus 0.74 using conventional clinical models, with an 8% reduction in prediction error. The model was able to accurately predict which patients were most likely to undergo delayed primary surgery, but it was not able to predict surgical cytoreductive outcomes.

In a similar manner, Yin et al. [24] analyzed the CT images of 757 patients from three hospitals, combining a total of 2271 images in order to predict the responses to NACT and distinguish between the different pathological types of OC using a multitask deep learning model based on multiperiod CT images. The multitask model yielded an AUC of 0.85 in predicting chemotherapy response in the validation set and a 9% increase in AUC using the multiperiod CT images. Upon multivariate logistic regression, peritoneal carcinomatosis and inguinal lymph node metastasis were found to be significantly associated with a higher response to NACT (*p* < 0.05). The multitask deep learning model, when combined with these clinical risk factors, achieved high performance in predicting NACT response with AUCs of 0.87 in the training set, 0.88 in the validation set, 0.86 in the prospective set, and 0.79 in the external set, which are extremely promising and warrant further investigation in clinical practice.

### 4.2. Prediction of the Outcomes of Cytoreductive Surgery

Parpinel et al. [25] conducted a systematic review of six articles with a total of 1899 patients to analyze the capacity of artificial intelligence in predicting the feasibility of complete cytoreduction in epithelial ovarian cancer patients. Using different AI methods like K-Nearest Neighbor, the XGBoost Model, Artificial Neural Networks, and the Decision Tree algorithm, the review found a predictive accuracy for surgical resection ranging from 65.8% to 77.7%, with a median AUC of 0.81; however, only two articles discussed the survival rate, with one reporting a 92% 5-year overall survival, while the other reported a 73% 2-year overall survival.

Piedimonte et al. [26] developed ML models to predict the outcome of cytoreduction in patients with advanced epithelial ovarian cancer using clinical, radiologic, and surgical parameters. Two models were created: one to predict optimal < 1 cm cytoreduction, and one to complete cytoreduction (0 mm RD). The model was developed using a cohort of 315 patients. The bootstrap Random Forest model had AUCs of 99.8% in the training set, 89.6% validation, and 89.0% in the test set. For outcome of no gross residual disease, the AUCs were 94.4%, 52%, and 84%, respectively. This is the first large study performed to predict the outcomes of cytoreduction; however, further studies are needed for prospective validation.

Similarly, Maubert et al. [27] verified the possibility of using a classification algorithm to predict the resectability of peritoneal carcinomatosis (PC) for OC patients who were eligible for cytoreduction surgery with hyperthermic intraperitoneal chemotherapy (HIPEC). Using a sample of 310 OC patients, the author demonstrated that Random Forest outperformed all models with an accuracy of 97.8% in predicting unresectability, which was determined based on the surgery outcome.

Shinagare et al. [15], with a total of 57 HGSOC patients that underwent cytoreductive surgery with surveillance abdominopelvic CT imaging, using a ten-fold cross-validation method with an L-SVM kernel ML strategy, found that the CA125 measurement was 62% accurate in predicting OC recurrence in addition to CT findings (*p* = 0.007).

Li et al. [29] assessed the ability of diffusion-weighted MRI (DW-MRI) combined with morphological characteristics in predicting the outcome of primary cytoreductive surgery in HGSOC patients. The morphological characteristics were divided into two categories: mass-like with a well-defined boundary, solid or cystic-solid mass, clear demarcations from surrounding tissues, and an association with lower DWI-PCI scores; versus infiltrative with a poorly defined boundary, soft tissue involvement with less clear demarcation, a higher extent of invasion into surrounding tissues, and an association with higher DWI-PCI scores. Using a cohort of 95 patients, the author reported an AUC of 0.824 for DWI-PCI to accurately predict surgical outcome as opposed to a clinicopathological model with an AUC of 0.811. Combining both models and using clinical factors like CA-125 and the amount of ascites yielded a higher AUC of 0.863. Thus, the combination model provides a reliable and promising non-invasive method to accurately predict cytoreductive outcomes in HGSOC patients.

Laios et al. [30] assessed the capacity of K-Nearest Neighbour (KNN) to predict the successful response of the complete cytoreduction in achieving an R0 resection in HGSOC patients using a pathological evaluation of the examined tissues and, in certain cases, a CT scan post-operation to confirm the absence of a residual tumour. Using a sample of 154 OC patients, KNN outperformed LR in accurately predicting an R0 resection with a mean accuracy of 65.8% vs. 63.4%, respectively.

### 4.3. Prediction of Platinum Resistance

Bi et al. [31] analyzed the data of 394 HGSOC patients using MRI-based radiomics to predict the response to platinum-based chemotherapy, using T2-weighted images, Contrast-enhanced T1-weighted images, and an apparent diffusion coefficient. Three models were performed to assess the best performance, comparing platinum-resistant and platinum sensitive groups. The habitat model demonstrated a superior performance when compared to the deep learning model and radiomics model, with AUCs of 0.710, 0.603, and 0.640, respectively, in predicting patients who will have recurred within 6 months of platinum-based treatment. Combining the habitat model with NACT as a clinical predictor yielded the highest AUC, 0.721, in predicting the response to platinum-based chemotherapy.

Bergstrom et al. [32] developed a deep learning AI platform to identify DNA repair deficiency (HRD) responsible for breast and ovarian cancer. The new model, DeepHRD, utilized Deep Neural Networks to achieve high accuracy in predicting responses to platinum chemotherapy using whole-slide H&E images. The model was initially conducted on breast cancer samples, and then transfer learning was applied on OC with a sample of 589. The capacity of DeepHRD to predict HRD status in ovarian cancer patients had an AUC of 0.81 using the digitalized H&E slides. The model predicted a median survival of 4.6 years based on HRD prediction, as opposed to the base model with no learning transfer (HR = 0.53 [0.26–1.07] 95% CI: q-value = 0.076). Thus, this model can be a promising tool, using HRD status as a predictor of survival.

Similarly, Zhuang et al. [33] evaluated deep learning models—DenseNet, ResNet18, and Swim Transformer—to predict platinum resistance in HGSOC patients using PET/CT images. Using data from 289 HGSOC patients, the model achieved an accuracy of 92.6%, positive predictive value (PPV) of 95.7%, negative predictive value (NPV) of 85.7%, sensitivity of 86.3%, and specificity of 96.1% in predicting platinum resistance. The study demonstrates a high potential of deep learning models to accurately predict platinum resistance compared to traditional methods and could help identify platinum-resistant patients early on the disease course to offer alternative targeted therapies and guide recommendations, such as surgery and systemic treatments.

Yi et al. [34] developed an SVM model with genomic data and pre-treatment CT images to predict platinum resistance in 102 patients. This model had the highest performance among all studies. Using a SULF-1 protein assay in addition to radiomics and clinicopathologic information, this model showed high classification efficiency, with an AUC of 0.99 in the training cohort, a sensitivity of 100.0%, and a specificity of 96.2. The AUC was 0.967 in the validation cohort, with a sensitivity of 100.0% and a specificity of 91.3%. This study shows that use of a combined model demonstrates significant clinical benefits compared to any model built with a single data source such as radiomics, clinicopathological models, or SNPs.

### 4.4. Prediction of Survival

Fotopoulou et al. [35] reported the performance of a novel radiomic biomarker, *Radiomic Prognostic Vector (RPV)*, in predicting progression-free survival (PFS) in 323 HGSOC patients undergoing cytoreductive surgery. The RPV was calculated using standardized algorithms following the segmentation of routine preoperative imaging of patients. Using the RPV score, patients with high RPV had a worse PFS compared to those with low RPV, with a median PFS of 18.1 months vs. 25.9 (*p* = 0.004, HR = 1.69; 95% CI:1.06–2.71; *p* = 0.038). In addition, patients with low RPV were 26% (*p* = 0.02) and 17% (*p* = 0.11) more likely to be progression-free at 2 and 3 years after surgery, respectively. This study highlights the potential of this novel biomarker in predicting relapse and progression-free survival in HGSOC patients.

Yang et al. [36] integrated DNA methylation and transcriptome data and identified 419 potential epigenetically regulated long non-coding RNAs. Based on the validation cohort, 186 patients were classified as high risk (*n* = 92) or low risk (*n* = 94). Patients in the low-risk group had a 5-year survival rate of 41%, while those in the high-risk group had a survival rate of 28%. The overall median survival time was 3.2 years for low-risk patients and 4.8 years for high-risk patients.

Enshaei et al. [37] studied survival data for 668 EOC patients. The ML models were compared to conventional statistical approaches, such as logistic regression, using different algorithms and classifiers. An ANN algorithm was used to predict survival and was more accurate (93%) than the LR models.

Zhao et al. [38] investigated the potential impact of genes associated with copper metabolism (CMRGs) on the prognosis and survival outcomes in OC. With a sample of 477 OC patients and 133 CMRGs, utilizing LASSO–Cox to establish CMRGs’ prognosis signature, the authors were able to divide genes in two groups: the low-risk group (LR), characterized by a low expression of CMRGs, and the high-risk group (HR), with higher expression of CMRGs based on copper metabolism risk score. The overall survival was better predicted in the low-risk group with an AUC for 5-year survival of 0.74 based on a better immune cell infiltration and response to immunotherapy (Tozasertib and Staurosporine), compared to the HR, where they were more sensitive to specific systemic therapy (Trametinib and Sinularin), making a tailored treatment more beneficial.

## 5. Discussion

Our study is the first to describe the role of machine learning and radiomics in predicting treatment responses in advanced ovarian cancer and biomarkers that may be used to predict improved survival. In this study, we pooled the results of machine learning/radiomics models to predict outcomes of neoadjuvant treatment and primary surgery, both of which had high performance (AUC 0.77 and 0.82, respectively), demonstrating that AI models can serve as a decision aid in clinical settings to help decide optimal treatment modality in patients with advanced ovarian cancer. Furthermore, some of the survival models had AUC up to 93% and overall performed much better than traditional statistical models, suggesting avenues for future research to improve precision in ovarian cancer.

### 5.1. What This Study Adds

The findings of the present study, albeit a small sample size, are very promising in regard to assisting predictions of treatment response in ovarian cancer. This is crucial, as it can assist the selection of patients for chemotherapy, surgery, targeted therapy, and ultimately improve survival in a disease with previous poor prognosis. In addition, extracting crucial data from pre-treatment images can be performed through radiomics and offer enhanced prediction outcomes.

In the prediction of NACT response, the models presented in this study performed over 80%; this is crucial and clinically significant when selecting patients for interval cytoreduction. The models included in our study also correlated radiomic analysis with chemoresponse scores and highlight that there may be pre-treatment predictors of chemoresponse which may facilitate decision making between surgery or targeted therapy. We previously published the KELIM score as one such predictor of chemoresponse, but radiomics has the ability to extract data from imaging and offer improved predictions [39].

Furthermore, we showed that ML/radiomics models improve the prediction of platinum-resistant patients compared to traditional statistics (with an AUC over 70%). Of note, Yi et al. reported an exquisitely high AUC in all test sets (over 95%) using a novel biomarker SULF-1, which may be further explored in the future. This is clinically meaningful, as novel targeted agents, such as antibody drug conjugates (ex; *mirvetuxumab*), have emerged as the new standard of care in platinum-resistant ovarian cancer. Using ML, radiomics, and novel biomarkers, these patients can be selected earlier in the course of their disease, and have improved survival and limited toxicity to futile lines of chemotherapy.

On the contrary, our group previously published a systematic review on the use of ML in endometrial cancer, and the algorithms did not perform better than traditional methods with regard to diagnosis and treatment [40]. Moreover, we also published an analysis of survival in high-grade endometrial cancer patients which also did not show improved results compared to traditional statistics [41]. We therefore concluded that in EC, ML algorithms may not play such an impactful role as in other fields of medicine. Thus, the current study on OC adds important information and further avenues to pursue the development of ML algorithms, particularly using radiomics, as most models have shown a very high performance (AUCs around 80%), which is higher than traditional statistics and objective physician assessments.

### 5.2. Results in the Context of the Published Literature

As only very few studies exist on this topic, our findings are in line with the reviews that were previously published. In addition, our group published an abstract on all ML algorithms in ovarian cancer [18]; however, the topic was too vast to include in a single manuscript, which is why we selected to focus on treatment outcome prediction, as this may improve survival and optimize patient selection.

A recent meta-analysis by Wang et al. [42] that used multiple ML algorithms to assess ovarian cancer patients’ response to platinum-based chemotherapy showed a pooled sensitivity and specificity of 0.89 and 0.79, respectively. With a more focused approach using solely radiomics, Zhuang et al. reported on a similar sensitivity of 86.3% and higher specificity of 0.96 [11]. In addition, Bi et al. and Bergstrom et al. have utilized MRI-based radiomics to predict responses to platinum-based chemotherapy [9,10]. Both studies highlight the potential of radiomics to accurately predict responses to treatment, thus offering more personalized therapeutic strategies to patients, and are presented in our Section 3 and tables.

Laios et al. used K-Neural Networks to predict the response of complete cytoreduction with a predictive accuracy of 66% [18]. This is quite low, and lower than the cited suboptimal cytoreduction rate of 15%. On the other hand, our group previously developed a machine learning algorithm using Random Forests and found a much higher prediction of optimal (<1 cm cytoreduction) with an AUC of 89.6% in the test set, while for the outcome of no gross residual, the AUC for the test set was 84%. Piedimonte et al. [26] and Parpinel et al. [25] have demonstrated the critical utility of machine learning, specifically in guiding the decision-making strategy for optimal cytoreduction, allowing for a greater outcome and reducing morbidity.

With regard to survival, there are only three published articles since 2021 using radiomics and biomarkers to predict 2-year and 5-year survival. Zhao et al. published a study demonstrating the capacity of LASSO to predict 5-year survival for ovarian cancer patients. Its model has demonstrated an AUC of 0.79 [22], while Parpinel et al. found greater prediction accuracy for the 5-year survival using radiomics of 0.91 [13].

### 5.3. Strengths and Limitations

Our study highlights a novel field of medicine to predict treatment response in high-grade ovarian cancer. This study synthesizes the available literature and highlights the promising results of ML and radiomics algorithms for treatment, offering a pathway to more guided and personalized treatment for HGSOC patients, potentially leading to improved outcomes.

The main the limitation of this study is the small sample size and the heterogeneity of the studies, which precluded a meta-analysis. This is explained by the limited number of publications that explore the use of radiomics in treatment prediction.

### 5.4. Future Directions

Our study confirms that ML and radiomics models are worth pursuing and validating in a prospective fashion, as they can truly lead to improved outcomes and ultimately to survival. Radiomics may offer an even better avenue by extracting important information from CT scans and diagnostic laparoscopy images to predict resectability, response to chemotherapy, and targeted therapy, and can lead to improved survival.

## 6. Conclusions

Our study finds an increasing use of ML and radiomics in ovarian cancer, particularly in the last 3 years. Specifically, when used for predictions of treatment outcomes, including chemotherapy and surgery, the models performed highly, with an AUC over 80%. The magnitude was lower in predicting platinum resistance (over 70%), but still clinically meaningful, and may be used to select patients for targeted therapies. Future prospective and validation studies warrant further investigation prior to widespread implementation.

## Figures and Tables

**Table 1:** Characteristics of the studies reporting Ml/radiomics algorithms for the prediction of treatment response and cytoreduction in patients with advanced ovarian cancer.

Author	Year of Publication	Country	Input	Outcome	Sample Size	Machine Learning Mode.	Sens.	Spec.	AUC
**Rundo et al. [22]**	2022	UK	CT images	NACT outcome	109	Elastic Net logistic regression	-	-	0.87
**Crispin-Ortuzar [23]**	2022	UK	Clinical parameters and CT images	NACT outcome	134	Elastic net, Support vector regressor, Random forest	-	-	0.78
**Yin et al. [24]**	2023	China	CT images	NACT outcome	757	CNN	-	-	0.85
**Parpinel et al. [25]**	2023	Italy/France	Clinical parameters and vaginal ultrasound/PET/CT images	Cytoreductive outcome/survival prediction	1899	k.NN, XGBoost Model ANN, MLDTA	-	-	0.81
**Piedimonte et al. [26]**	2022	Canada	Clinical parameters	Cytoreductive Surgery outcome	315	Gradient-boosted tree model, Random Forest	-	-	0.896
**Maubert et al. [27]**	2019	France	CT images	Cytoreductive Surgery outcome	310	Support Vector, Random Forest, Conditional Tree, Simple classification	-	-	0.978
**Shinagare et al. [28]**	2018	USA	CT images	Cytoreductive Surgery outcome	57	Support Vector	-	-	0.62
**Li et al. [29]**	2022	China	MRI images and Clinical parameters	Cytoreductive outcome/survival prediction	95	DW-MRI	-	-	0.863
**Laios et al. [30]**	2020	UK	CT images	Cytoreductive Surgery outcome	154	KNN	-	-	0.658
**Bi et al. [31]**	2023	China	MRI/clinical parameters	Platinum Resistance	394	K-Nearest Neighbour, LASSO	0.8	0.697	0.721
**Bergstrom et al. [32]**	2023	USA	Histopathological slides	Platinum Resistance	589	CNN	-	-	0.81
**Zhuang et al. [33]**	2023	China	PET/CT images	Platinum Resistance	289	SE-SPP-DenseNet model	0.863	0.961	-
**Yi et al. [34]**	2021	China	CT images	Platinum Resistance	102	LASSO, Random Forest	1.0	1.0	0.967

Legend: NACT: neoadjuvant chemotherapy. Sens: sensitivity. Spec: specificity.

## Data Availability

Data are available upon request from the journal.
